# Investigating factors influencing decision-making around use of breastmilk substitutes by health care professionals: a qualitative study

**DOI:** 10.1186/s13006-024-00656-7

**Published:** 2024-07-10

**Authors:** Maisha Islam, Dourra Assani, Serine Ramlawi, Malia SQ Murphy, Kameela Miriam Alibhai, Ruth Rennicks White, Alysha LJ Dingwall-Harvey, Sandra I Dunn, Darine El-Chaâr

**Affiliations:** 1 School of Epidemiology and Public Health, University of Ottawa, Ottawa, Canada; 2 Faculty of Medicine, University of Ottawa, Ottawa, Canada; 3 Clinical Epidemiology Program, Ottawa Hospital Research Institute, Ottawa, Canada; 4 Department of Obstetrics, Gynecology & Newborn Care, The Ottawa Hospital, Ottawa, Canada; 5 Better Outcomes Registry and Network Ontario, Children’s Hospital of Eastern Ontario, Ottawa, Canada; 6 School of Nursing, University of Ottawa, Ottawa, Canada; 7 Department of Obstetrics & Gynecology, University of Ottawa, Ottawa, Canada

**Keywords:** Exclusive breastfeeding, Baby-Friendly initiative, Breastmilk substitutes, Supplementation rates, Breastfeeding support, Postnatal care

## Abstract

**Background:**

Breastfeeding is recognized as the gold standard of infant feeding and nutrition. The World Health Organization recommends exclusive breastfeeding (EBF) of infants for the first 6 months of life. A variety of factors may impact breastfeeding practices in-hospital which may continue after hospital discharge, such as the use of breastmilk substitutes (BMS). The Baby-Friendly Initiative (BFI), which aims to promote and support breastfeeding practices, established a target rate of 75% for EBF from birth to hospital discharge. Currently, this target is not being met at The Ottawa Hospital (TOH), indicating there is room for improvement in EBF rates. The purpose of this study is to explore health care professionals (HCP) decision-making around use of BMS and identify factors that drive the use of BMS with and without medical indications.

**Methods:**

In this qualitative study, semi-structured interviews were conducted with HCPs within TOH from January to June 2022. All participants had experience in maternity or postpartum care and were probed on factors influencing use of BMS at this institution. Interview transcripts were coded using an inductive approach.

**Results:**

A total of 18 HCPs were interviewed including physicians, midwives, lactation consultants, and registered nurses. Multilevel barriers influencing the use of BMS were categorized into patient, HCP, and institution-level factors. Subthemes that emerged ranged from parental preferences, training differences amongst HCPs, to budget and staffing issues. Over half of HCPs were prepared to answer questions on EBF and were familiar with the BFI. Although most were supportive of this institution receiving BFI designation, a few providers raised concerns of its impact on parents who would like to supplement.

**Conclusions:**

Several modifiable factors influencing decision-making for use of BMS were identified. These findings will be used to inform unit leads, help identify effective strategies to address modifiable barriers, and develop tailored breastfeeding supports to improve EBF rates.

**Supplementary Information:**

The online version contains supplementary material available at 10.1186/s13006-024-00656-7.

## Background

The World Health Organization (WHO) and Health Canada recommends initiation of breastfeeding within the first hour after birth and exclusive breastfeeding (EBF) for the first 6 months of life [1, 2]. Breastfeeding, referred to as the feeding of human milk directly from the breast or expressed milk through an alternative method (i.e. bottle or cup), offers a multitude of immunological, physiological, and psychological benefits for mothers and children [3–6]. In 1991, the WHO and UNICEF launched the Baby Friendly Hospital Initiative (BFHI) to protect, promote and support breastfeeding in maternity services [7]. The Breastfeeding Committee for Canada (BCC) established the Baby Friendly Initiative (BFI) Guideline which is a Canadian adaptation of the BFHI breastfeeding standards. The BFI Guideline recommends that supplementation of breastmilk with substitutes, such as infant formula, should be for documented medical indications [8]. In Canada, 91% of mothers initiate breastfeeding, however, approximately 15% stop before the infant is one month old and only 35% of mothers exclusively breastfeed until their infant is six months of age [9]. For the province of Ontario, there are similar trends in breastfeeding with an initiation rate of 92% and 36% of parents exclusively breastfeeding for at least 6 months [9]. Both provincial and national rates are significantly lower than the WHO’s Global Nutrition Target of 50% EBF in the first 6 months by 2025 (and 70% by 2030) [10, 11]. 

A variety of factors may influence infant feeding decisions at birth and after hospital discharge such as social and cultural norms, insufficient resources to support breastfeeding, lactation management issues, maternity legislation regarding return to work, and lack of breastfeeding education amongst parents and health care professionals (HCPs) [12–14]. The promotion of breastmilk substitutes (BMS) is another key factor hindering breastfeeding practices. The commercial milk formula industry threatens breastfeeding by influencing both HCPs and other consumers to support BMS use through methods such as digital marketing, advertisements in health care facilities, provision of free formula samples, and sponsorships at public events [15]. In 1981, the International Code of Marketing of BMS was adopted by the WHO to address the harmful consequences associated with BMS use and to protect breastfeeding practices worldwide [16]. The Code and subsequent World Health Assembly Resolutions highlights the responsibilities of governments, health care systems and workers, and BMS manufacturers in the labelling and marketing of BMS products as well as providing objective and accurate information on infant feeding [16]. Although adoption of the Code does not guarantee that it is legislated, as of 2022, 144 of 194 (74%) WHO Member States have enforced legal measures to implement at least some of the provisions of the Code [17]. Canada and the United States remain some of the few high-income countries amongst the remaining 50 countries to have implemented no legal measures at all.

HCPs play a critical role in the initiation and overall trajectory of breastfeeding during an infant’s first six months of life. A multi-country study consisting of 300 interviews with a variety of HCPs found that HCPs were the main source of education on infant feeding practices and that HCPs’ recommendations strongly influenced women’s breastfeeding decisions [15]. The same study found that although HCPs’ views on breastfeeding have become more positive in recent years, it was still common practice for HCPs to recommend BMS in many countries [15]. Furthermore, a cross-sectional study from Canada exploring reasons for in-hospital non-medically indicated (NMI) supplementation in infants found that the top determinants of NMI supplementation included: no previous breastfeeding experience, negative first impressions of breastfeeding and receiving breastfeeding advice from a physician [18]. Another study assessing HCP perceptions of the BFI at a pediatric institution in Ottawa, Canada demonstrated that even though 75% of the 651 participants believed the implementation of the BFI to be important or very important, only slightly over one-third felt it was important or very important that all hospital personnel receive breastfeeding training [19]. These findings collectively suggest that HCPs can influence parents’ decision to breastfeed their infants and the success of in-hospital BFI initiatives.

Understanding the factors contributing to use of BMS is fundamental to improving EBF rates globally and in Canada. The Ottawa Hospital (TOH) is a tertiary-care academic hospital in Ottawa, Canada serving over one million people across Eastern Ontario and recorded over 6,000 births in 2022. Currently, EBF rates at TOH are below the recommended target of 75% set by the BFI. The lack of adherence to BFI recommendations demonstrates an evidence-based practice gap and highlights the importance of exploring the various determinants of breastfeeding and decision-making around the use of BMS at this institution. This study aimed to identify modifiable factors that contribute to the use of BMS among HCPs from birth to hospital discharge.

## Methods

### Study design and participant sample

This was a cross-sectional qualitative study using semi-structured interviews conducted at TOH, which has two main sites and provides obstetrical and neonatal care in the form of prenatal clinics, birthing units, special care nurseries, and a neonatal intensive care unit to patients in the Ottawa-Gatineau region. All HCPs who provide maternity and/or postpartum care were eligible to participate. Participants were recruited by e-mail invitation or participation was discussed in-person with HCPs on eligible care units. All participants provided verbal consent. Participants were informed prior to the interview that their participation would not affect their employment at the hospital and that all transcripts would be de-identified and analyzed anonymously. Recruitment continued until inductive thematic saturation was achieved and there was no longer emergence of new themes [20].

### Data collection

Semi-structured interviews were conducted from January to June 2022 using a 19-question interview guide (see Additional file 1). First, HCPs were asked a series of demographic questions regarding their employment history, experience with the obstetric population, and their experiences providing breastfeeding support and BMS to patients. Next, HCPs were probed on their knowledge and practices regarding breastfeeding protocols and the use of BMS as well as their perceptions of institutional infant feeding policies and EBF training offered. Lastly, HCPs were asked about their prior breastfeeding training (i, e., quantity and quality of training received), attitudes toward breastfeeding/BFI, and knowledge of institutional and community resources available to support patients who want to exclusively breastfeed. The interviews were iterative such that questions were added, removed, or changed as they were being conducted and analyzed to account for new themes or challenges that arose with certain questions. The interview guide was reviewed by a focus group including a maternal-fetal medicine specialist, a lactation consultant (LC), nurses with obstetrical training, and an epidemiologist. Individual interviews were conducted virtually and transcribed verbatim using Microsoft Teams [21] and all transcripts were manually reviewed for accuracy. The average interview length was approximately 35 min, with interviews ranging from 24 min to approximately 1 h.

### Data analysis

The transcripts of each interview were imported to NVivo 11™ [22] for thematic analysis. Conventional content analysis, an inductive coding approach, was used to code and analyze the data to allow themes to emerge from textual data [23]. A study team member (M.I) coded the data from each transcript and then reviewed and verified the coding scheme and data within each code with the larger team. Initial codes were then grouped into subthemes and broader themes by finding common underlying meaning between these categories and were discussed between study team members to build consensus regarding study findings.

## Results

A total of 18 interviews were conducted with HCPs including physicians, midwives, nurses, and LCs. HCPs’ experience in providing maternity or postpartum care ranged from less than 1 year to 25 years, with multiple providers having previous experience outside of the participating hospital. Demographic characteristics are summarized in Table 1.

**Table 1:** Characteristics of participating health care professionals

*Participant Characteristics (* *n* * = 18)*	No. and Frequency (%)
**Type of Health Care Professional**	
Maternity/postpartum providers^A^	13 (55)
Infant providers^B^	5 (45)
**Years of experience in maternity/postpartum care at participating hospital**	
0–4 years	12 (66)
5–10 years	3 (17)
10 + years	3 (17)
**Total years of experience in maternity/postpartum care**	
0–4 years	7 (39)
5–10 years	6 (33)
10 + years	5 (28)

^A^ Maternity/postpartum providers refer to lactation consultants, midwives, and nurses from the labour and delivery and postpartum units

^B^ Infant providers refer to nurses and a physician from the neonatal intensive care unit

Several HCPs were employed at other institutions throughout Canada prior to their employment at TOH, with some of these locations being BFI-accredited. The factors contributing to the provision of BMS by HCPs from birth to discharge were multidimensional and encompassed a variety of challenges which we categorized into three main themes: patient, HCP, and institution-level factors (Table 2).

**Table 2:** Factors influencing institutional exclusive breastfeeding rates and use of breastmilk substitutes

Main Themes	Subthemes	Initial Codes
**Patient-level factors**	Medical indications	Infant hypoglycemia
		Infant weight loss
		Premature birth
		Low milk supply
	Physical factors	Maternal fatigue / weakness
		Mother-infant dyad separation after birth
	Parental request	Lack of prenatal breastfeeding education
		Patient’s previous breastfeeding experience
		Partner / familial influence
**Health care professional level factors**	Training	Inconsistent / outdated training
		Unfamiliarity with institutions’ protocols / policies
	HCP personal attributes	Personal attitudes / beliefs on breastfeeding
		Knowledge on exclusive breastfeeding & Baby-Friendly Initiative
**Institution-level factors**	Budget & staffing shortages	Increased workload on staff
		Limited access to lactation consultants
	BMS documentation	Vague documentation of BMS use on hospital system

### Patient-level factors

Patient-level factors were directly related to the mother or infant and were categorized into three subthemes:1) medical indications, 2) physical factors, and 3) parental request. *Medical indications* for supplementation were the most frequently reported reasons for providing BMS from birth to hospital discharge. Hypoglycemia, weight loss of > 10% from birthweight, and premature birth were the top 3 infant medical indications for supplementation reported by HCPs. Low milk supply was the most noted maternal reason for use of BMS. Additionally, reasons for the use of BMS may be physical in nature, but not necessarily be medically indicated. Maternal fatigue or weakness after birth was the most common *physical factor* contributing to the provision of BMS, followed by separation of the mother-infant dyad. Fatigue or weakness was mostly perceived by HCPs, which lead to recommendation of BMS use, but at times was also patient-reported and the request for BMS would be from the patient directly.

*“ I think it’s left to the nurse and the mom to really decide whether it’s time to have formula or not, and if mom’s had a really rough delivery, like she’s already weak and tired, and if you have a nurse that’s not sold 100% on breastfeeding, then it’s just easy to say you’ve had a really rough delivery here’s some formula.”(P7)*.

HCPs also highlighted *parental request* for BMS as a key barrier to EBF from birth to discharge. Gaps in prenatal breastfeeding education among mothers was found to be a key driver of parental requests, as demonstrated by the following quote:

*“Most mums come in and they really don’t know, they had their pre-set ideas, but they haven’t really had education during their pregnancy about the benefits of breastfeeding in general and the risks of not breastfeeding.” (P1).*

Almost all HCPs agreed that most mothers intended to exclusively breastfeed following birth, with a few intending to combination feed. Despite having the intention to exclusively breastfeed, HCPs also noted that most mothers weren’t prepared to do so. Previous experience with breastfeeding was a key topic mentioned where HCP’s highlighted multiparous mothers were more likely to exclusively breastfeed successfully, while primiparous mothers required additional encouragement and support.

*“The mothers who breast fed in the past know what worked last time and what didn’t work last time. They know that breastfeeding takes time […] it doesn’t work as easy for some than others. They know that some positions worked better for them last time. And they know the amount of time and work they’ll have to invest for it to be done successfully.” (P7)*.

Lastly, HCPs perceived familial influence as another contributing factor to parental request of BMS. Opinions given by the patient’s maternal figures and the patient’s partner appeared to influence patient decisions for using BMS.

*“Because their mother bottle fed them and the grandmother bottle fed them, all they know is bottle feeding and they already bought their Good Start* [a common brand of commercial milk formula available in Canada] *and their bottles at home. So they come [to the hospital] with the notion to do this regardless.” (P4)*.

### HCP-level factors

HCP-level factors included two subthemes: (1) variations in HCPs’ training, and (2) personal attributes, such as beliefs and knowledge of EBF. Under the *training* subtheme, most HCPs reported they felt prepared to address patient questions about breastfeeding. However, a few reported that information provided to patients by other HCPs sometimes clashed with their own training and education, thereby influencing their confidence to provide breastfeeding support. Likewise, HCPs mentioned that patients would comment on inconsistencies in the information shared by different providers, which may be attributed to differences in career-level.

*“One thing I noticed that a lot of our parents comment on is that they received different information from different health care providers on breastfeeding in particular. But I think a lot of that comes from just maybe nurses being at different stages in their careers. So, some nurses may have received their training quite a long time ago but there are some key things that should be consistent throughout everyone’s teaching that they’re doing for parents.” (P5)*.

There were considerable differences amongst HCPs’ responses when questioned about training provided by TOH on EBF and the appropriate use of BMS. Almost half of the providers reported that specific training wasn’t provided by the institution. The remaining providers mentioned that breastfeeding training was provided in the form of a ‘Breastfeeding Day’ during orientation, and that nurse educators circulated updates related to breastfeeding education through email. HCPs identified a range of external training sources they sought out to improve their own breastfeeding education such as participating in courses or workshops at other hospitals, virtual workshops and modules, conferences, and personal readings.

The subtheme, HCP *personal attributes*, was further broken down into HCP attitudes and beliefs on EBF and knowledge of EBF and BFI. This topic was explored when asked if HCPs initiated conversations with patients who specifically requested substitutes without medical indication. Most HCPs reported they focused on gentle probing to identify specific reasons for patient BMS requests and provided education on the benefits of breastfeeding and current evidence-based practice, while respecting the patient’s decision. In contrast, upon patient request of substitutes, a couple of HCPs indicated that they typically wouldn’t question or follow-up on the benefits of breastfeeding and would instead provide BMS without discussion.

*“I’m a firm believer that ‘fed is best’ and I don’t believe that exclusively breastfed is best, I think that puts a lot of societal pressures on mum. And if they don’t feel like they’re meeting those pressures, that’s how they get you know, into postpartum depression and anything like that, so I believe that fed is best. If a patient looks to me and says, ‘I think I’d like to supplement’ I think that’s fine.” (P3)*.

There was a mixed response to questions focused on HCPs’ knowledge and support of EBF and TOH acquiring BFI accreditation. Over half of HCPs reported they were prepared to answer questions about EBF. Likewise, although more than half of HCPs were familiar with and supportive of BFI, some raised concerns about the initiative, with one provider being completely against obtaining the designation.

*“I would absolutely be supportive of [BFI], yes. As long as the patients are still respected further and their wishes. I know the initiative is great […] and having that accreditation is a huge positive, but at some points, it can be very alienating to patients who don’t want to or for whatever reason can’t breastfeed.”(P8)*.

*“I’ve heard very negative things about it. You sign a consent acknowledging the risks of formula. So there’s just, like, a lot of formula shaming and you need a doctor’s order, so it takes away the nurses ability to use their judgment.”(P9).*

### Institution-level factors

Institution-level factors contributing to poor EBF rates were divided into two subthemes including *budgeting and staffing shortages* and *BMS documentation*. The most frequently cited institution-level factor amongst HCPs were budget and staffing shortages in the department and the increased workload on staff, which hindered HCPs from providing adequate breastfeeding support. The small number of LCs on staff and their limited working hours were considered a significant contributor to low EBF rates as many patients who require specialized breastfeeding care are not able to receive a consultation until the next working day or risked being discharged before receiving LC support.

*“When you have someone who’s struggling with breastfeeding, it can take a lot of nurses’ resources. So, when you have multiple other patients, it can be easier just to hand off a bottle of formula instead of doing the educational piece of ‘OK, why do you want formula?’ ‘How can I help you to meet your aforementioned goals instead of just handing it to you?’ So, it does come down to staffing numbers a lot.” (P11)*.

Additional institution-level factors reported among HCPs include a vague documentation system that poorly captures BMS administration by hospital staff. This issue was further exacerbated when HCPs skipped documenting reasons for using BMS altogether.

*“… it’s really easy to just grab formula and give it to [the infant] and there’s no accountability, so you can’t see what nurse is getting it. You’re supposed to document on [electronic medical chart] why you’ve provided a supplement, but it’s very very rare that someone will actually document why they did.” (P18)*.

## Discussion

In this study, we explored HCP decision-making around use of BMS at TOH and identified factors contributing to their use with and without medical indications. Our findings suggest that several multilevel barriers influenced the provision of BMS at the patient, HCP, and institution-level.

### Patient-level factors

Patient-level factors such as medical indications, physical factors, and parental requests contributed to the use of BMS. These findings are similar to other studies investigating in-hospital formula supplementation including a study conducted across hospitals in the United States that reported medical indications, maternal request/preference/feelings, and lactation management-related issues as the top three most common reasons for formula supplementation reported by hospital staff [24]. Furthermore, our findings were consistent with several of the top reasons for maternal request of BMS reported by a public health unit in Ottawa, Canada including: medical conditions of mother or infant, inconvenience/fatigue/lack of time/finding breastfeeding too demanding, and milk supply concerns [25]. Maternal complaint of not producing enough milk, often referred to in the literature as “self-reported insufficient milk” (SRIM), has been frequently cited as a common reason for introduction of BMS [26, 27] and overall breastfeeding cessation [28]. Avoiding in-hospital formula supplementation, improving breastfeeding counselling, and increasing maternal breastfeeding self-efficacy are integral in protecting against SRIM and unnecessary BMS use [26, 27].

Additionally, parental request for BMS may be attributed to poor prenatal breastfeeding education and preparation for newborn care, which was a recurring theme in our interviews. A qualitative study conducted in Northern California investigating decision-making around maternal request for formula reported similar results with inadequate preparation for breastfeeding and using formula as a solution to breastfeeding problems as key reasons for in-hospital supplementation [29]. Furthermore, a systematic review assessing the effectiveness of prenatal education on breastfeeding outcomes found that caregivers participating in prenatal programs had higher breastfeeding uptake and knowledge, increased positive attitudes towards breastfeeding, as well as better self-efficacy [30].

### HCP-level factors

Additionally, HCP-related factors associated with BMS use was another main theme that emerged from our interviews. Clinicians’ beliefs and attitudes on breastfeeding and recommendations for use of BMS are significantly associated with in-hospital breastfeeding initiation and EBF [31, 32]. In our findings, while most HCPs were highly supportive of EBF and indicated they would review the benefits of breastfeeding with parents who requested BMS, others stated they would provide a BMS without question. Most HCPs didn’t want mothers to feel pressured to breastfeed or evoke feelings of guilt for wanting to supplement with formula. “Formula-shaming” is a controversial topic in the literature and consequent negative effects on the mother have been explored. A recent systematic review revealed that formula feeders experienced guilt more often than breastfeeding individuals and that external guilt was most associated with HCP influence [33]. 

Additionally, inconsistency in training amongst staff may further exacerbate this issue as it facilitates confusion and miscommunication among HCPs, and thereby to the patient as well. Key points HCPs highlighted was that some of the training may be outdated or inconsistent, or that they were unfamiliar with hospital protocols and policies. The development of skills and knowledge needed to support a breastfeeding individual is essential for ensuring breastfeeding in-hospital and continuity after discharge. One study showed that resident physicians who had higher participation in a breastfeeding education program were more likely to have better breastfeeding rates amongst their patients [31]. Similarly, another study found that the odds of breastfeeding were greater in hospitals where there was breastfeeding education for new employees, nurses received breastfeeding education in the past year, and there was a written breastfeeding policy [34]. The same study found that the time invested by hospitals in staff training is proportionally related to improved breastfeeding outcomes [34]. This can be compared to the BFI’s *Breastfeeding Success Steps*, which highlights the importance of having a written breastfeeding policy routinely communicated to HCPs and to ensure all HCPs have the knowledge and skills to implement the policy [8]. Thus, along with updated training, it is key that institutions facilitate straightforward communication of policies to staff to bridge any gaps between current knowledge and clinical practice.

### Institution-level factors

Lastly, we identified institutional-level factors that challenged HCPs’ ability to provide adequate breastfeeding support to their patients and promote potential use of BMS. Resource limitations, including budget and staffing shortages, was a dominant subtheme identified in this study. Most HCPs highlighted how low staff numbers hindered the amount of staff time spent available to provide breastfeeding support to patients, coupled with the need to monitor multiple patients for the duration of their shift. This is similar to studies conducted in other institutions which found that nurse staffing shortages were major barriers to implementing the BFI’s *Ten Steps to Successful Breastfeeding*, particularly due to insufficient time in providing adequate breastfeeding support [35, 36]. HCPs also highlighted the need for additional LC coverage, especially after regular working hours such as evenings, weekends, and holidays. A recent systematic review demonstrated that lactation consultants play a key role in improving breastfeeding initiation and EBF rates [37]. LCs are often referred to cases with medical indications and may not have time to speak with other patients, which may also contribute to the use of BMS if effective support isn’t provided by other staff members to patients in need. HCPs also noted there may be incomplete documentation of BMS use on the hospital system that may contribute to vague breastfeeding data.

Beyond factors that we identified at the patient, HCP, and institution levels specific to our study site, there may be additional external factors influencing overall infant feeding decisions. Many conceptual models have been developed to describe the multilevel interactions between the determinants of breastfeeding, such as the 2023 *Lancet breastfeeding series framework* [38]. Along with mother-infant factors, the authors highlight the impact of different structural factors (i.e., marketing and political economy of commercial formula) and settings (i.e., workplace and employment) on infant feeding practices. For example, although the topic of BMS marketing was not a major theme identified in our study, it is important to note how the commercial milk formula industry has long lasting impacts on breastfeeding practices. Currently, our institutional policies prohibit marketing of BMS within the hospital and to parents. This may contribute to why this issue was not raised during the interviews. However, Canada remains one of several countries that have no legal measures to regulate marking of BMS as suggested by the International Code of Marketing of BMS [17]. Thus, future studies investigating the effect of BMS marketing on breastfeeding practices at a local, provincial, and national scale are warranted. Additionally, maternity employment legislations are also key determinants of parents’ infant feeding decisions [39]. Studies have shown that extended paid maternity leave are associated with positive breastfeeding outcomes including higher breastfeeding initiation and duration rates [40–42]. Currently, the province of Ontario supports up to 63 weeks of parental leave which promotes retention of breastfeeding beyond 6 months. However, this leave is unpaid, and parents may only receive benefits through the federal government depending on specific eligibility criteria.

### Implications

**Fig. 1:**
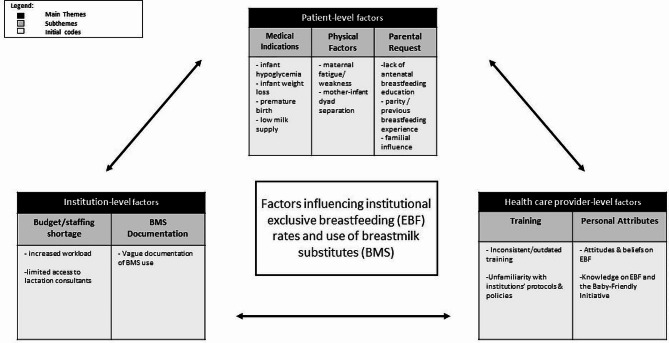
Factors influencing institutional exclusive breastfeeding (EBF) rates and use of breastmilk substitutes (BMS)

Our findings highlight several modifiable barriers to EBF at TOH. Although we identified three separate categories contributing to EBF rates and use of BMS, understanding the interconnectedness between all categories is critical in improving breastfeeding outcomes in this institution. For example, institutional policies and training may undermine an HCPs knowledge and skills on providing breastfeeding support which may further influence the mother’s confidence and ability to breastfeed their infant. This may ultimately contribute to BMS use. Likewise, shortage of funding and staff may increase the stress and workload of HCPs which may lead to inadequate in-hospital breastfeeding support for the mother and subsequent use of BMS that may continue post-hospital discharge. Thus, developing multilevel interventions is critical to maintaining long-term change. Figure 1 portrays our initial codes, subthemes, and main themes and the interactions between these themes.

Many HCPs proposed strategies that could help overcome current barriers to increase EBF rates and reduce use of BMS. Developing and distributing resources that promote prenatal breastfeeding education among patients may address issues related to misinformation and negative beliefs and attitudes about breastfeeding. HCPs also suggested that more frequent and updated training could help limit differences in clinical practice between staff members and improve overall skills and knowledge in providing breastfeeding support. This can be addressed through developing annual electronic learning modules for this population or obligatory workshops delivered throughout the year. Budgeting and staffing shortages may not be easily addressed. However, identifying breastfeeding champions from existing staff may improve patient access to lactation support. Additional suggestions included developing resources such as a virtual breastfeeding support platform, walk-in clinic hours with LCs, and establishing a connected hospital-to-community perinatal care system to support patients’ post-hospital discharge. Lastly, another suggestion to reduce BMS use included implementing a mandatory note in patients’ medical chart. This would help capture in-depth information and support regular audits on the frequency and circumstances for supplementation at TOH. Although our findings were similar to those of many other international studies, we advise further research to be conducted on this topic in Canada to better understand the targeted interventions required to improve national breastfeeding rates and decrease use of BMS. Further research is also required to understand how lack of legal measures on BMS marketing may impact infant feeding practices throughout Canada. To achieve the WHO’s goal of 70% EBF in the first 6 months of life by 2030, implementing provisions from the International Code of Marketing of BMS is a pivotal first step.

### Strengths and limitations

This study offers valuable insight into HCP decision-making around use of BMS; however, limitations in our study exist. First, there were a few cases where it was difficult to differentiate between situations that were medically indicated versus NMI as such situations were not further elaborated on during interviews. For example, low milk supply and premature birth may be medically indicated in circumstances defined by the BCC such as if due to primary glandular insufficiency or if the infant was born at less than 32 weeks of gestation [8]. Likewise, separation of the mother-infant dyad was listed as a physical factor but may be due to medically indicated reasons. In addition, the voluntary nature of the study may contribute to selection bias as participants may be more interested in issues surrounding breastfeeding and the use of BMS. As the sample was only a subset of all eligible HCPs, our findings are not representative of the beliefs and attitudes of all eligible clinical staff at TOH. Self-reporting bias is also a potential limitation as HCPs may report information that may be inaccurate and reflects the HCP’s recollection of the situation or event. Social desirability bias must also be considered as HCPs may answer in a manner that is positively regarded by the researchers and the public but is not reflective of their own views.

Strengths of this study include the diversity of HCPs interviewed for the study across their clinical roles and extent of experience in maternity and infant care. In addition, a variety of methods were used to mitigate potential biases. The study’s semi-structured design enabled flexibility when conducting interviews where emerging themes may be probed with new questions. Likewise, the interviewer is a research trainee at the institution but is not directly affiliated with the department and does not hold a position of authority relative to the participants to ensure an impartial interview process. Lastly, the results of this study will be used to inform a larger department-wide survey to investigate the determinants of BMS use and low EBF rates in this institution. The results of our research can be used to drive similar studies both regionally and nationally.

## Conclusion

Our findings highlight that the factors contributing to the use of BMS from birth to discharge are multifactorial but modifiable and may be best addressed using targeted interventions. Important steps to minimize unnecessary use of BMS include improving institutional HCP training by updating and standardizing training, providing reliable prenatal breastfeeding education to patients, and allocating resources toward a designated breastfeeding support provider or LC to aid other maternity care staff. Implementation of these strategies necessitates collaboration between hospital staff, departmental leaders, and higher-level hospital executives to establish sustainable change.

### Electronic supplementary material

Below is the link to the electronic supplementary material.

Supplementary Material 1

## Data Availability

The data will not be distributed due to confidential information.

## Abbreviations

BCC: Breastfeeding Committee for Canada
BFI: Baby-Friendly Initiative
BFHI: Baby-Friendly Hospital Initiative
BMS: Breastmilk substitute
EBF: Exclusive breastfeeding
HCP: Health care professional
LC: Lactation consultant
NMI: Non-medically indicated
TOH: The Ottawa Hospital
WHO: World Health Organization

## References

[1] World Health Organization. Counselling of women to improve breastfeeding practices [Internet]. 2018 [cited 2022 Mar 7]. https://www.who.int/publications/i/item/9789241550468. 30933442

[2] Health Canada. Nutrition for healthy term infants: Recommendations from six to 24 Months [Internet]. 2023 [cited 2023 Dec 7]. https://www.canada.ca/en/health-canada/services/canada-food-guide/resources/nutrition-healthy-term-infants/nutrition-healthy-term-infants-recommendations-birth-six-months/6-24-months.html.

[3] Victora CG, Bahl R, Barros AJD, França GVA, Horton S, Krasevec J (2016). Breastfeeding in the 21st century: epidemiology, mechanisms, and lifelong effect. Lancet.

[4] Public Health Agency of Canada. 10 great reasons to breastfeed your baby [Internet]. 2020 [cited 2023 Dec 7]. https://www.canada.ca/en/public-health/services/publications/healthy-living/10-great-reasons-to-breastfeed-your-baby.html.

[5] Krol KM, Grossmann T (2018). Psychological effects of breastfeeding on children and mothers. Bundesgesundheitsblatt Gesundheitsforschung Gesundheitsschutz.

[6] Tucker Z, O’Malley C (2022). Mental health benefits of breastfeeding: a literature review. Cureus.

[7] World Health Organization. Baby-friendly hospital initiative: revised, updated and expanded for integrated care [Internet]. 2009 [cited 2023 Dec 7]. https://www.who.int/publications/i/item/9789241594950. 23926623

[8] Breastfeeding Committee for Canada. Baby-Friendly Implementation Guideline [Internet]. 2021 [cited 2022 Sep 13]. https://breastfeedingcanada.ca/wp-content/uploads/2021/05/BFI-Implementation-Guideline-May-19.pdf.

[9] Public Health Agency of Canada. Canada’s Breastfeeding Progress Report 2022 [Internet]. 2022 [cited 2023 Dec 7]. https://health-infobase.canada.ca/src/data/breastfeeding/PHAC%20-%20Breastfeeding%20Report%202022.pdf.

[10] World Health Organization. Global Nutrition Targets 2025: Policy Brief Series [Internet]. 2014 [cited 2023 Dec 7]. https://iris.who.int/bitstream/handle/10665/149018/WHO_NMH_NHD_14.2_eng.pdf?sequence=1.

[11] World Health Organization. UNICEF. The extension of the 2025 Maternal, Infant and Young Child nutrition targets to 2030 [Internet]. 2019 [cited 2024 Feb 16]. https://data.unicef.org/resources/who-unicef-discussion-paper-nutrition-targets/.

[12] Balogun OO, Dagvadorj A, Anigo KM, Ota E, Sasaki S (2015). Factors influencing breastfeeding exclusivity during the first 6 months of life in developing countries: a quantitative and qualitative systematic review. Matern Child Nutr.

[13] Office of the Surgeon General (US). Centers for Disease Control and Prevention (US), Office on Women’s Health (US). Barriers to breastfeeding in the United States. In: The Surgeon General’s Call to Action to Support Breastfeeding [Internet]. Rockville; 2011 [cited 2023 Dec 12]. https://www.ncbi.nlm.nih.gov/books/NBK52688/.

[14] Mason F, Rawe K, Wright S. Superfood for Babies: How overcoming barriers to breastfeeding will save lives [Internet]. London; 2013 [cited 2023 Dec 12]. https://www.savethechildren.org/content/dam/global/reports/health-and-nutrition/baby-superfood-asia.pdf?vanityurl=BF-Report.

[15] World Health Organization. UNICEF. How the marketing of formula milk influences our decisions on infant feeding [Internet]. Geneva; 2022 [cited 2023 Dec 12]. https://iris.who.int/bitstream/handle/10665/352098/9789240044609-eng.pdf?sequence=1.

[16] World Health Organization. International Code of Marketing of Breast-milk Subsitutes [Internet]. Geneva. 1981 [cited 2023 Dec 11]. https://iris.who.int/bitstream/handle/10665/40382/9241541601.pdf?sequence=1.

[17] World Health Organization. Marketing of breast-milk substitutes: National implementation of the International Code, Status Report 2022 [Internet]. Geneva; 2022 [cited 2023 Dec 11]. https://www.unicef.org/media/120071/file/Marketing%20of%20Breast%E2%80%91milk%20Substitutes%20Status%20Report%202022.pdf.

[18] Bonia K, Twells L, Halfyard B, Ludlow V, Newhook LA, Murphy-Goodridge J (2013). A qualitative study exploring factors associated with mothers’ decisions to formula-feed their infants in Newfoundland and Labrador, Canada. BMC Public Health.

[19] Pound C, Ward N, Freuchet M, Akiki S, Chan J, Nicholls S (2016). Hospital staff’s perceptions with regards to the Baby-Friendly Initiative. J Hum Lact.

[20] Saunders B, Sim J, Kingstone T, Baker S, Waterfield J, Bartlam B (2018). Saturation in qualitative research: exploring its conceptualization and operationalization. Qual Quant.

[21] Microsoft. Microsoft Teams. 2022.

[22] Lumivero, NVivo. QSR International Pty Ltd; 2022.

[23] Hsieh HF, Shannon SE (2005). Three approaches to qualitative content analysis. Qual Health Res.

[24] Bookhart LH, Anstey EH, Kramer MR, Perrine CG, Reis-Reilly H, Ramakrishnan U (2022). A nation-wide study on the common reasons for infant formula supplementation among healthy, term, breastfed infants in US hospitals. Matern Child Nutr.

[25] Ottawa Public Health. Infant Feeding in Ottawa 2012 to 2014 [Internet]. 2015 [cited 2023 Dec 13]. https://www.ottawapublichealth.ca/en/reports-research-and-statistics/resources/Documents/infant_feeding_2015_en.pdf;46.

[26] Pierro J, Abulaimoun B, Roth P, Blau J (2016). Factors associated with supplemental formula feeding of breastfeeding infants during postpartum hospital stay. Breastfeed Med.

[27] Huang Y, Liu Y, Yu XY, Zeng TY (2022). The rates and factors of perceived insufficient milk supply: a systematic review. Matern Child Nutr.

[28] Ricci C, Otterman V, Bennett TL, Metcalfe S, Darling E, Semenic S (2023). Rates of and factors associated with exclusive and any breastfeeding at six months in Canada: an analysis of population-based cross-sectional data. BMC Pregnancy Childbirth.

[29] DaMota K, Bañuelos J, Goldbronn J, Vera-Beccera LE, Heinig MJ (2012). Maternal request for in-hospital supplementation of healthy breastfed infants among low-income women. J Hum Lact.

[30] Kehinde J, O’Donnell C, Grealish A (2023). The effectiveness of prenatal breastfeeding education on breastfeeding uptake postpartum: a systematic review. Midwifery.

[31] Holmes AV, McLeod AY, Thesing C, Kramer S, Howard CR (2012). Physician breastfeeding education leads to practice changes and improved clinical outcomes. Breastfeed Med.

[32] Ramakrishnan R, Oberg CN, Kirby RS (2014). The association between maternal perception of obstetric and pediatric care providers’ attitudes and exclusive breastfeeding outcomes. J Hum Lact.

[33] Jackson L, De Pascalis L, Harrold J, Fallon V (2021). Guilt, shame, and postpartum infant feeding outcomes: a systematic review. Matern Child Nutr.

[34] Li CM, Li R, Ashley CG, Smiley JM, Cohen JH, Dee DL (2014). Associations of hospital staff training and policies with early breastfeeding practices. J Hum Lact.

[35] Alakaam A, Lemacks J, Yadrick K, Connell C, Choi HW, Newman RG (2018). Maternity nurses’ knowledge and practice of breastfeeding in Mississippi. MCN Am J Matern Child Nurs.

[36] Cunningham EM, Doyle EI, Bowden RG (2018). Maternity nurses’ perceptions of implementation of the ten steps to successful breastfeeding. MCN Am J Matern Child Nurs.

[37] Patel S, Patel S (2016). The effectiveness of lactation consultants and lactation counselors on breastfeeding outcomes. J Hum Lact.

[38] Pérez-Escamilla R, Tomori C, Hernández-Cordero S, Baker P, Barros AJD, Bégin F (2023). Breastfeeding: crucially important, but increasingly challenged in a market-driven world. Lancet.

[39] Baker P, Smith JP, Garde A, Grummer-Strawn LM, Wood B, Sen G (2023). The political economy of infant and young child feeding: confronting corporate power, overcoming structural barriers, and accelerating progress. Lancet.

[40] de Lauzon-Guillain B, Thierry X, Bois C, Bournez M, Davisse-Paturet C, Dufourg MN (2019). Maternity or parental leave and breastfeeding duration: results from the ELFE cohort. Matern Child Nutr.

[41] Mirkovic KR, Perrine CG, Scanlon KS (2016). Paid maternity leave and breastfeeding outcomes. Birth.

[42] Chai Y, Nandi A, Heymann J (2018). Does extending the duration of legislated paid maternity leave improve breastfeeding practices? Evidence from 38 low-income and middle-income countries. BMJ Glob Health.

