# Supplementary material for: Investigating factors influencing decision-making around use of breastmilk substitutes by health care professionals: a qualitative study
Source: Int Breastfeed J. 2024 Jul 10;19:48. doi: 10.1186/s13006-024-00656-7 (PMC11234533; doi:10.1186/s13006-024-00656-7)
Supplement: Supplementary file 1 — Supplementary Material 1 [file 13006_2024_656_MOESM1_ESM.pdf]

Additional File 1: Interview Guide

Demographic Questions

1. **Can you please describe your position in the Department of Obstetrics, Gynecology and Newborn Care at [participating institution]? (PROBE: staff nurse, midwife, manager, clinical educator, clinical facilitator, lactation consultant….)**

2. **Have you held any other positions within the department?**

3. **How long have you been working in the department?**
    - In your current position?
    - In previous position?

4. **How many years have you been working with obstetrical patients?**
    - How many years with postpartum patients, specifically?

5. **Do you have any other relevant experience and/or training in providing breastfeeding support within or outside of [participating institution]?**

Key Interview Questions

6. **Based on your experience, do most women who plan on breastfeeding , intend to exclusively breastfeed, or do they come to hospital undecided or intending to combination feed following birth (breastfeed and use human milk substitute (e.g. formula))?**

7. **For the women intending to exclusively breastfeed, do you feel they are well prepared prior to birth?**

Possible probing topics:

- What are some key differences between primiparous and multiparous mothers?
- How realistic do you feel individuals are about their breastfeeding journey?
- What are some common questions that mothers ask you about exclusive breastfeeding and/or use of human milk substitutes (formula)?
    - Do you feel prepared / knowledgeable to answer questions about exclusive breastfeeding?
    - What resources do you provide your patients on exclusive breastfeeding? How available are these resources to you? How often do you estimate you receive questions about use of human milk substitutes?

8. **Walk me through what strategies you use to support or promote exclusive breastfeeding among your patients.**

<u>Possible probing topics:</u>

- What differences in strategies do you use for mothers who have never breastfed and mothers who are experienced?
- From your experience, are there some strategies that work better than others?
    - Can you describe them to me?
- Are there any tips and tricks that you provide to your patients?

9. **Under what circumstances or for what reasons might you as a [insert HCP type] introduce use of a human milk substitute to feed an infant between birth and discharge?**

<u>Possible probing topics:</u>

- Do you discuss the use of human milk substitutes with the parent(s)?
    - If so, what do you tell them?
    - Does this occur before or after feeding?
- Have you noticed an increase or decrease in use of human milk substitutes over the course of your work experience with postpartum patients (including medical indications)?

10. **When a health care provider orders a human milk substitute for medical reason, do you feel that all cases are justifiable and that they are following best evidence-based practices?**

11. **Are there circumstances where you might introduce human milk substitutes when it is not medically indicated? Can you describe them to me? (e.g., convenience of care, request of the parent, etc.)**

<u>Possible probing topics:</u>

- Where do requests for human milk substitutes typically come? (e.g., nurse, midwife, physician, parent)?
- How often do you estimate you use human milk substitutes when it is not medically indicated? (E.g., per day? per week?)
- What types of human milk substitutes do you provide at [participating institution]? (e.g formula or water)
- For what reasons might a patient request the use of human milk substitutes when it is not medically indicated?
    - How often do you estimate this happens?
    - Approximately how long after delivery does this typically occur?
- If an individual requests a human milk substitute for the baby, what kind of conversation do you have, if any, with the individual?

12. **Have you received any training regarding the appropriate use of human milk substitutes and promoting exclusive breastfeeding practices through [participating institution]?**

Possible probing topics:

- From where are you receiving this training? How often (one time only/annually)?
- What topics were included in this training?
- How do you apply the content of the training into clinical practice?
- Do you feel confident in the training you have received from [participating institution] to be able to help a new parent with: Breastfeeding? Answering breastfeeding related questions? Providing anticipatory guidance about breastfeeding?
- If no training is provided, is it expected that healthcare providers have prior knowledge on breastfeeding and helping the newborn latch?

13. **Have you sought out any training on your own regarding the appropriate use of human milk substitutes and promoting exclusive breastfeeding practices?**

Possible probing topics:

- From where are you receiving this training? How often (one time only/annually)?
- What topics were included in this training?
- How do you apply the content of the training into clinical practice?
- Do you feel confident in your training you have received from external sources to be able to help a new parent with: Breastfeeding? Answering breastfeeding related questions? Providing anticipatory guidance about breastfeeding?

14. **To your knowledge, are there any tools/policies/procedures at [participating institution]to encourage exclusive breastfeeding?**

Possible probing topics:

- What breastfeeding supports are available for breastfeeding individuals at [participating institution]and what are the referral criteria for an individual to access a lactational consultant?
- Where is the formula stored on the labor & delivery and post-partum floors?
- Can you describe to me the procedures for requesting or accessing human milk substitutes (formula) on the labor & delivery and post-partum floors?

15. **Are there currently any resources (including resources available through [participating institution] and the community) that are available in the antepartum period to encourage breastfeeding after birth? [for antenatal HCP only]**

16. **Are there ways in which you think [participating institution] could better support individuals to breastfeed exclusively?**

Possible probing topics:

- Are there sufficient resources provided to individuals prior to delivery about breastfeeding practices?
- What challenges do you as a [insert HCP] face in supporting breastfeeding individuals?
- Are there ways in which you think the department could help reduce the use of human milk substitutes when there are no medical indications?

17. **Are there any protocols or practices within the hospital that you believe prevent or enable [participating institution] from achieving higher rates of exclusive breastfeeding? If yes, what are they?**

Possible probing topics:
For example, if parents have already decided they want to combination feed their newborn, are HCP's encouraged to inform the individual on the advantages of exclusive breastfeeding?

18. **Are you familiar with the Baby Friendly Initiative (BFI)? If yes, would you be supportive of [participating institution] obtaining BFI accreditation?**

Possible probing topics:

- How do you think this could benefit [participating institution]?

19. **Is there anything else that you would like to share with me regarding how [participating institution] or the department could help increase exclusive breastfeeding rates or reduce the use of human milk substitutes from birth to discharge?**
